# Wall-to-Lumen Ratio Estimation in Adaptive Optics Retinal Imaging: Evaluation and Comparison

**DOI:** 10.1167/tvst.15.10.26

**Published:** 2026-10-16

**Authors:** Radim Kolar, Roman Jakubicek, Eva Orsagova, Jan Odstrcilik, Franziska G. Rauscher

**Affiliations:** 1 Department of Biomedical Engineering, Faculty of Electrical Engineering and Communication, Brno University of Technology, Brno, Czech Republic; 2 Institute for Medical Informatics, Statistics, and Epidemiology, Leipzig University, Leipzig, Germany; 3 Germany Medical Informatics Center - Department of Medical Data Science, Leipzig University Medical Center, Leipzig, Germany

**Keywords:** adaptive optics (AO), retinal imaging, vascularity, wall-to-lumen ratio (WLR), retinal artery

## Abstract

**Purpose:**

Retinal arteriolar changes reflect systemic microvascular alterations associated with cardiovascular risk, including hypertension. The retinal arteriolar wall-to-lumen ratio (WLR), obtained by adaptive optics (AO) imaging, is a promising noninvasive biomarker of early vascular damage. Its clinical utility is limited by inter- and intra-grader variability, lack of standardized protocols, and dependence on image quality, boundary definition, and measurement method. Standardized, objective WLR quantification is therefore needed to minimize observer bias and improve reproducibility.

**Methods:**

We compared point-based and region-based manual WLR estimation using 60 high-resolution AO images from 35 healthy subjects. Two experienced graders performed five point-based measurements per image, yielding one median WLR, followed by manual artery wall and lumen segmentation, yielding one WLR value. Inter-grader variability, method agreement, and sampling-location requirements were evaluated using statistical and Bland–Altman analyses.

**Results:**

Point-based assessment showed good inter-grader agreement, with a relative mean difference of 4.5% (Wilcoxon test, *P* < 0.05). Region-based assessment produced significantly lower WLR values than the point-based method (*P* < 0.01), with relative mean differences of 19.9% and 26.1% for graders A and B, respectively. Inter-grader relative mean difference was 6.2%, comparable to the point-based method. Approximately 10 measurement positions reduced the estimation error to about 5%; fewer positions increased error. Image quality and vessel-border visibility affected WLR variability.

**Conclusions:**

WLR estimation is influenced by measurement approach, grader experience, image quality, and sampling density. Although region-based assessment is more comprehensive, it is time-consuming. For clinical point-based assessment, at least 10 clearly visible arterial locations are recommended to minimize estimation error. Standardized protocols are essential for reliable WLR quantification.

**Translational Relevance:**

For standardized manual WLR assessment in AO retinal images, we recommend measuring only clearly visible vessel segments, using double-sided measurements whenever possible, avoiding bifurcations, crossings, and adjacent vessels, obtaining ≥12 measurements per artery, excluding the upper and lower deciles, and reporting the median of ≥10 remaining measurements as the final WLR.

## Introduction

The retina provides a unique and invaluable platform for noninvasive, rapid, and repeated in vivo visualization and assessment of photoreceptor distribution and microvascular structures.^1^ Retinal arterioles exhibit alterations analogous to those observed in cerebral and peripheral arterioles under hypertensive conditions, suggesting that retinal arteriolar abnormalities reflect structural and functional microvascular changes in other end-organ tissues.^2^^,^^3^ Since the seminal work of Keith et al.^4^ in 1939, numerous studies have confirmed the prognostic relevance of advanced retinal vascular abnormalities in predicting cardiovascular mortality.^5^^,^^6^ However, whereas the prognostic significance of advanced retinopathy is well-supported,^2^^,^^7^ the relationship between early retinal vascular abnormalities and cardiovascular risk stratification remains less firmly established. Methodological limitations are thought to account for the inconsistency in the evidence linking early retinal vascular changes with cardiovascular risk.^2^^,^^8^ Recent research efforts have focused on developing innovative image processing method in the adaptive optics (AO) retinal imaging field (e.g. image stitching,^9^^,^^10^ cone detection,^11^^,^^12^ vascularity analysis,^12^ etc.), which are connected with enhancing the accuracy and reliability of extracted biomarkers. Among the various parameters, one of the most promising biomarkers is the wall-to-lumen ratio (WLR), which allows the detection of vascular changes, for example, in hypertension. These retinal changes have been shown to correspond to changes seen in the subcutaneous small arteries.^13^

The ability to measure and analyze WLR noninvasively represents a new frontier in ophthalmology, offering a potential biomarker for cardiovascular risk assessment^1^^,^^14^ including assessing systemic vascular health and risk.^15^ WLR is particularly relevant in diseases that affect the microcirculation, as it can reveal early structural alterations in the retinal microvasculature indicative of the initial stages of vascular remodeling.^16^ Age, arterial hypertension, and body mass index are among the factors that have been found to significantly influence WLR.^17^^,^^18^ An increase in WLR is often associated with these conditions, suggesting a correlation between systemic health problems and changes in the microvascular structure of the retina. Our previous work has also documented a WLR increase during pregnancy,^19^ highlighting its potential for assessing pregnancy-related cardiovascular adaptations.

WLR is an innovative biomarker of both retinal and systemic vascular health, showing significant promise as a noninvasive tool for the early detection of vascular changes. Although increasing clinical evidence supports its potential, surprisingly little is known about its measurement accuracy and error estimation. There are only a few studies describing the intra- or inter-observer variability of WLR extraction. According to a study by Meixner and Michelson^16^ that utilized an AO retinal camera for measuring WLR, intra-observer variability was 6% ± 0.9 (average ± standard deviation [SD]) for arteriolar wall thickness and 2% ± 0.2 for arteriolar wall thickness plus vessel lumen. The work of De Ciuceis et al.^20^ showed similar variability in WLR intra-observer (3.25% ± 1.40) or inter-observer (7.05% ± 4.59) variability.

The challenges in WLR estimation are primarily related to image quality, methodological variability, and lack of standardization. Accurate delineation of the inner and outer vessel borders depends heavily on image quality, which can be compromised by ocular media opacities (e.g. cataracts) or poor fixation in patients with certain conditions. Measurement errors may also result from misidentification of vessel boundaries, particularly in small arterioles with subtle luminal changes. In addition, WLR estimation is strongly influenced by the chosen methodology for manual assessment—whether point-based or region-based—and by the number of measurement locations analyzed along the vessel, both of which can differ between studies and affect the final WLR value. Despite recent advances, no universally accepted protocols currently exist for WLR evaluation using AO. Variability in imaging systems, analysis software, and operator expertise further contribute to inconsistencies across studies. Thus, in this work, we focus on the WLR estimation error more deeply and provide suggestions regarding the number of measurement positions.

## Methods and Data

### Approaches for WLR Calculation

To determine the WLR from retinal imaging data, the analysis was performed on annotated images using two distinct methods: a point-based approach and a region-based approach. Both techniques were applied and compared as part of this study. The overall processing pipeline is illustrated in Figure 1. In the point-based approach, WLR estimation was derived from points manually placed on the inner and outer artery walls, with point placement performed by the grader such that the corresponding measurement lines were perpendicular to the artery axis rather than generated automatically. This method reflects the conventional technique commonly used in clinical practice and is represented by the upper branch of the schematic in Figure 1. In contrast, the region-based approach used manual segmentation of the arterial lumen (grey segments, Fig. 2) and vessel walls (white segments, Fig. 2) to generate binary masks for WLR estimation. The segmentation was performed in the Medical Imaging Interaction Toolkit (MITK), developed at the German Cancer Research Center. These masks were further refined using morphological operations. A vessel skeleton was then extracted, and perpendicular lines were automatically defined for each skeleton point to locate the lumen and wall boundaries and compute WLR values. The number of lines is automatically defined depending on the number and size of detected walls, which typically is in the range of several hundred such lines per sampled artery. This approach, shown in the lower branch of Figure 1, follows similar geometric principles to the point-based method but leverages detailed segmentation for more comprehensive analysis.

**Figure 1.**
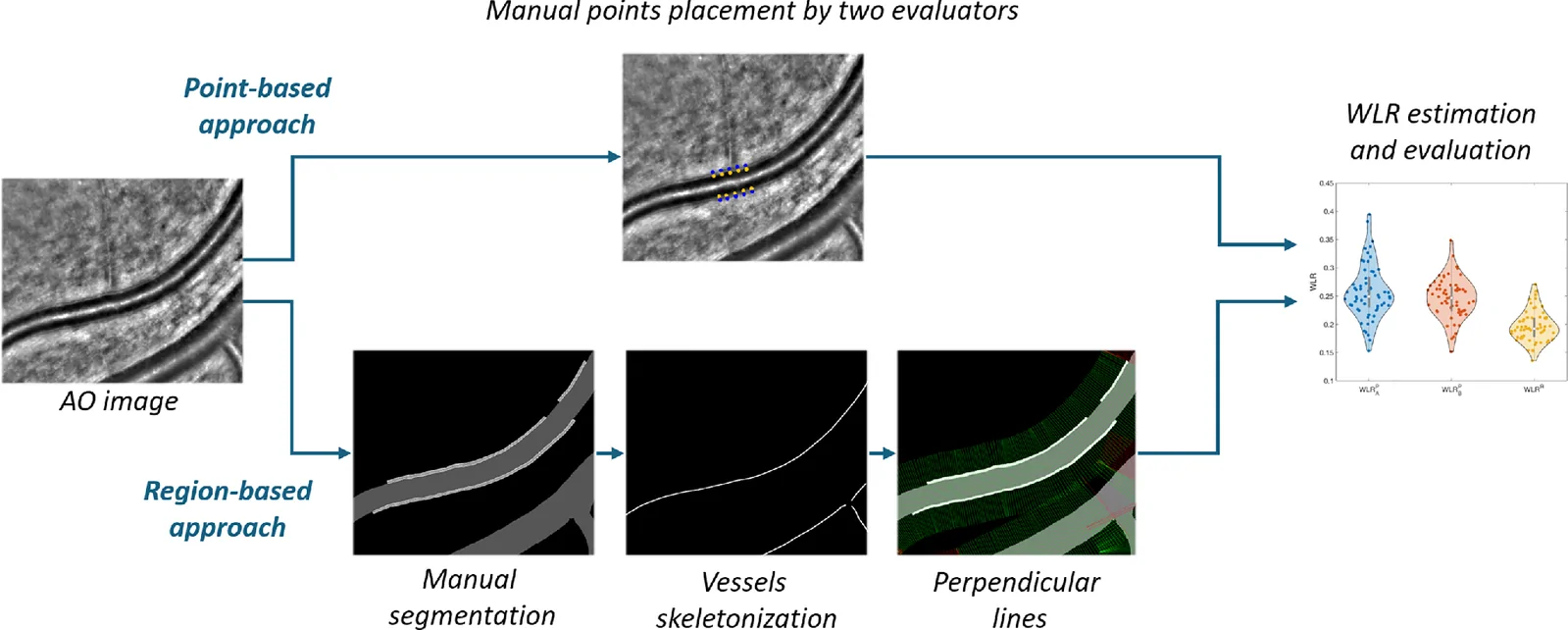
Illustration of processing pipeline: Scheme representing two methodologies of WLR estimation and their evaluation described in this work. The upper part depicts the point-based approach, whereas the lower part refers to the region-based approach. Corresponding detailed figures are provided in the respective section of this work. Details of presented steps are shown in subsequent figures and corresponding text.

**Figure 2.**
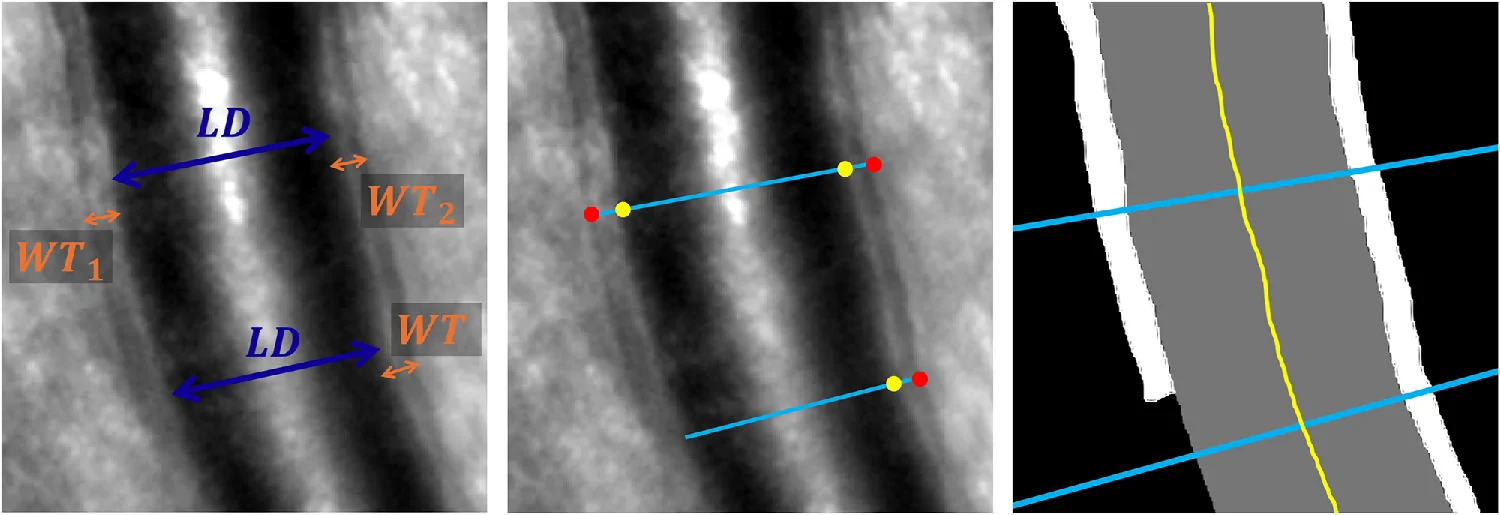
Illustration of the WLR measurement using wall thickness (WT) and lumen diameter (LD) for a double-sided or single-sided case to WLR estimation. The acquired image is depicted on the *left* with lumen diameter (LD) and wall thickness (WT) illustrated for both double-sided and single-sided options schematically. The *middle image* displays the WLR estimation using the point-based approach original image with manually placed points for two different example locations on the *straight blue line* (*red* for the outer boundary and *yellow* for the inner boundary). The *right image* illustrates the WLR estimation using the region-based approach – the segmentation of the artery and its walls is depicted (*white color* represents the wall and *gray color* the inner part of the artery), whereas the *blue lines* represent perpendicular directions to algorithmically detection artery skeleton (*yellow curve*).

Regardless of the approach used, the distance between the inner points on either side represents the lumen diameter (LD) and the distance between the inner and outer point defines wall thickness (WT), as shown in Figure 2. The value of WLR can be calculated in two different ways, depending on whether the artery wall is recognizable on both sides or on one side alone (see Fig. 2). For the double-sided calculation, we utilized both WTs (WT1 and WT2):

$$\begin{array}{c} WLR=\;\frac{WT_{1}+WT_{2}}{LD}, \end{array}$$

and in single-sided case, we calculated the WLR using the wall thickness from only one side (WT):

$$\begin{array}{c} WLR=\;2\frac{WT}{LD} \end{array}$$

### Experimental Data and Manual Annotation

For this study, 60 retinal images were analyzed from 35 subjects with no reported ocular disease, see Table 1. Participants were imaged at Leipzig University Hospital, Leipzig, Germany. Typically, one to two images per subject were acquired approximately one disc diameter from the optic nerve head. Image acquisition was performed using the rtx-1 retinal camera (Imagine Eyes, France), which uses flood-illumination adaptive optics to capture high-resolution en face images of the retina under near-infrared illumination (850 nm). The system corrects ocular aberrations in real time using a wavefront sensor and a deformable mirror, allowing visualization of individual cone photoreceptors and retinal microvascular structures. For each eye, 40 short-exposure frames (<10 ms) were acquired and subsequently averaged after motion correction by the device's integrated software to enhance image quality. The final averaged images were then exported using the built-in export tool as image file in PNG format.

**Table 1.** Basic Characteristics of the Dataset

No. of Images	No. of Subjects	No. of Right Eyes	No. of Left Eyes	No. of Men	No. of Women	Age Range, Y	Mean Age, Y
60	35	38	22	6	29	15.0–59.4	33.5

In the point-based approach, each image in the dataset was annotated independently by two expert graders. The graders first placed two points along the inner arterial boundary on opposite sides of the vessel and connected them with a straight line, see Figure 2. The outer arterial boundary was then marked along this line, either on both sides or only on one side, depending on the visibility of the arterial wall. For each image, five WLR measurements were obtained, typically along the same arterial segment or another branch of the same artery. An example of this procedure is shown in Figure 3 (left image). In clinical practice, artery wall evaluation is typically performed manually using proprietary ophthalmic software; however, in this study, we implemented a custom annotation tool in MATLAB (version 9.13.0, R2022b), which provided full control over the annotation process and data export.

**Figure 3.**
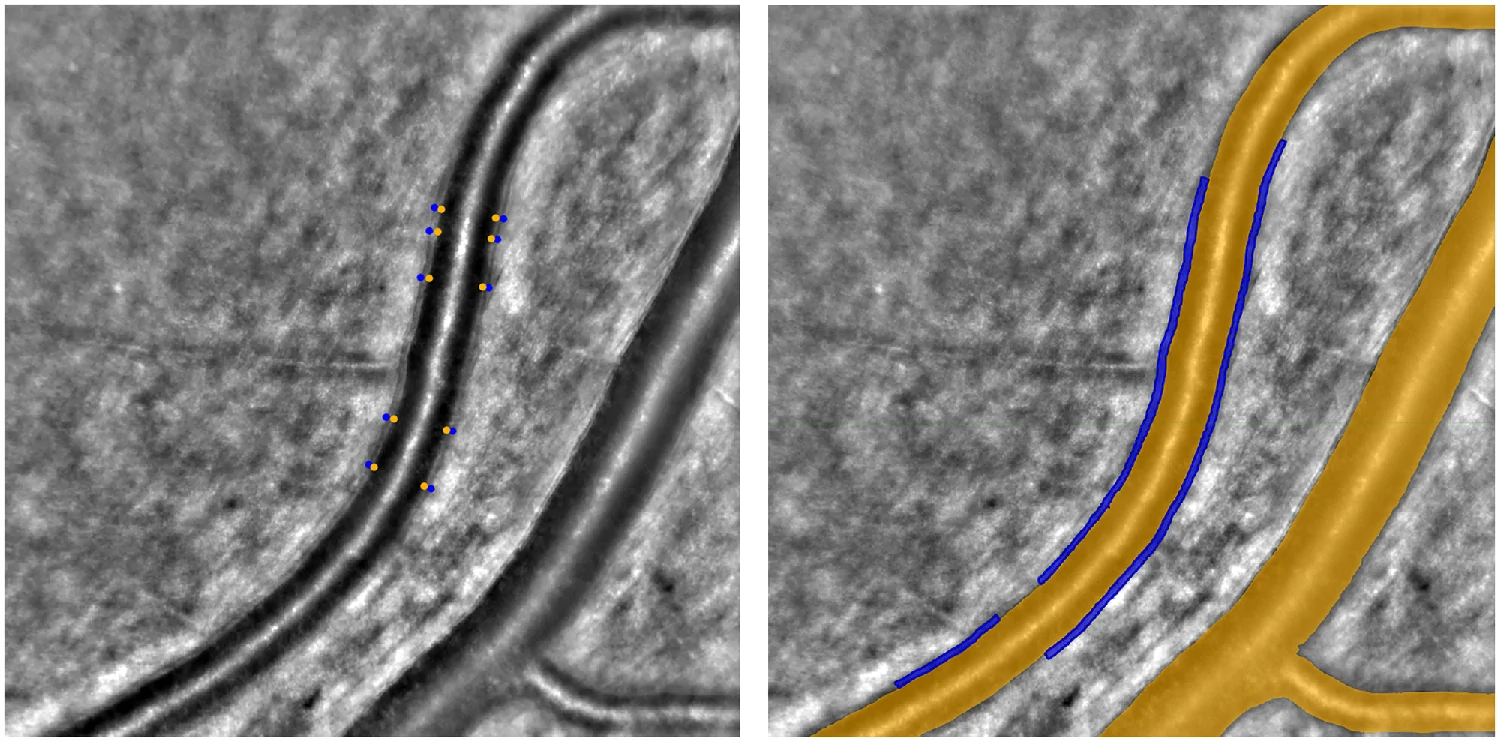
*Left* *image*: The original image with manually placed points for five different locations is shown (*blue* for the outer boundary and *yellow* for inner boundary). *Right* *image*: The original image with manual segmentation of the arterial lumen and the wall performed in Medical Imaging Interaction Toolkit software is depicted (*blue color* represents walls and *yellow color* represents the inner part of the arteries).

The second WLR assessment approach was performed in a region-based manner. Here, each grader segmented 30 images, amounting to 60 images in total. Thus, only one segmentation per image is available in this case. This manual annotation was carried out by the open-source software MITK (German Cancer Research Center) and an example of this segmentation is shown in Figure 3 (right image). Consequently, basic image processing operations were used to extract the central curve of the artery, define several local perpendicular lines, and compute WLR values.

We standardized the WLR measurement procedure for AO retinal images for both point-based and region-based approaches in the following way. Measurements were performed only at locations where the arteriolar wall and lumen boundaries were sharply defined, excluding blurred or out-of-focus regions. Thus, for point-based and region-based approaches, the expert graders were asked to manually mark or segment the artery walls in the images only at locations where these walls are sharp and visually recognizable. This is because the depth of field of the imaging device is limited and typically only part of the vessel appears sharp in the image. Blurred parts were ignored, as they do not carry any useful information about the wall structure. Double-sided measurements were obtained whenever both inner and outer wall boundaries were visible on both sides of the vessel, whereas single-sided measurements were used only when one vessel wall could not be reliably delineated. Measurement locations were distributed along the entire analyzable length of each arteriolar segment while avoiding vessel bifurcations, crossings, and regions where adjacent arteries or veins were in close proximity to the target vessel. The graders were instructed to avoid the locations close to vessel crossings or bifurcations, because WLR measurement can be influenced by the anatomic conditions and hemodynamics. Both graders have several years of comparable experience with image evaluation and segmentation, including AO images, as well as with the software used. During the segmentation process image adjustments to facilitate labeling are encouraged, for example, graders were free to zoom in on the images or adjust their contrast or both contrast and brightness on the monitor, as needed.

### Evaluation Strategies

Several comparisons were performed to investigate whether there is a statistical difference between point-based and region-based strategies and also whether there are differences between the graders. Furthermore, we focused on the reliability of the point-based approach to provide an answer to the question: “How many locations should be selected to get a reliable estimation of the WLR?” This is highly important for clinical practice because this point-based approach is currently typically used in various clinical and research settings, and it is implemented in the device software.

#### Point-Based and Region-Based Approach Comparison

We computed WLR for the images using the point-based approach as a median of five manually placed points. This value is denoted as $WLR_{p}^{A}$ and $WLR_{p}^{B}$, respectively, for the two graders, A and B. Then, we computed a median value of WLR from all possible locations determined from manually segmented artery walls (region-based approach), denoted as $WLR_{r}^{}$. Nevertheless, before calculating the median, we removed the upper and lower deciles of WLR values, as there are typically several tens to hundreds of these values. This increases the robustness of this estimate. We then used Bland–Altman plots to investigate the variability of the deviations of both approaches and graders.

When comparing different approaches or graders, we calculated both the absolute and relative differences in WLR. The relative difference was defined as the absolute WLR difference divided by the mean of the two WLR values being compared (from either the two approaches or the two graders).

#### Analysis of Reliability of Point-Based Approach Based Using the Region-Based Approach

Reliability analysis uses segmented images to extract final WLR values for a varying number of randomly selected positions. We used a standard random sampling algorithm to estimate the WLR in this scenario. In short, we started with one position and continued with two, three, up to the number determined by the size of the segments in the image (usually between 100 and 200). For each number of positions (*n*), we computed the WLR as a median. This sampling was repeated hundreds of times to obtain reliable information about the WLR value dependence on the number of sampling points. The relative error Δ*WLR_n_* was calculated as follows:

$$\begin{array}{c} \Delta {\mathrm{WLR}}_{n}=100\frac{{\mathrm{WLR}}_{n}-{\mathrm{WLR}}_{global}}{{\mathrm{WLR}}_{global}}\;\left[\%\right], \end{array}$$

where *WLR_n_* represents the WLR value calculated as an average for *n* locations and *WLR_global_* represents the WLR value estimated from a whole manually segmented image, considering maximum number of locations.

#### Bland–Altman Analyses and Statistical Analysis

Bland–Altman analysis was performed to assess agreement, first, between the two expert graders for the point-based approach and, second, for each grader agreement was assessed between the two measurement approaches. The mean bias and the 95% limits of agreement were calculated, together with their 95% confidence intervals (CIs). Systematic bias was evaluated using a paired *t*-test, and proportional bias was assessed by linear regression of the measurement differences against the corresponding means. Statistical significance was defined as *P* < 0.05.

## Results

### Region-Based and Point-Based Approach Comparison

#### Main Statistical Description and Comparison

The violin plots of the distribution of WLR values for the two used approaches are shown in Figure 4. Because the normality assumption was violated for $WLR_{p}^{A}$ values, we applied the Wilcoxon rank-sum test. The test rejected the null hypothesis for all comparisons, indicating that the differences between the corresponding median WLR values are statistically significant. The violin plots suggest larger separation between distribution of WLR values between the region-based approach with respect to both point-based WLR values. The main statistical characteristics are summarized in Table 2.

**Figure 4.**
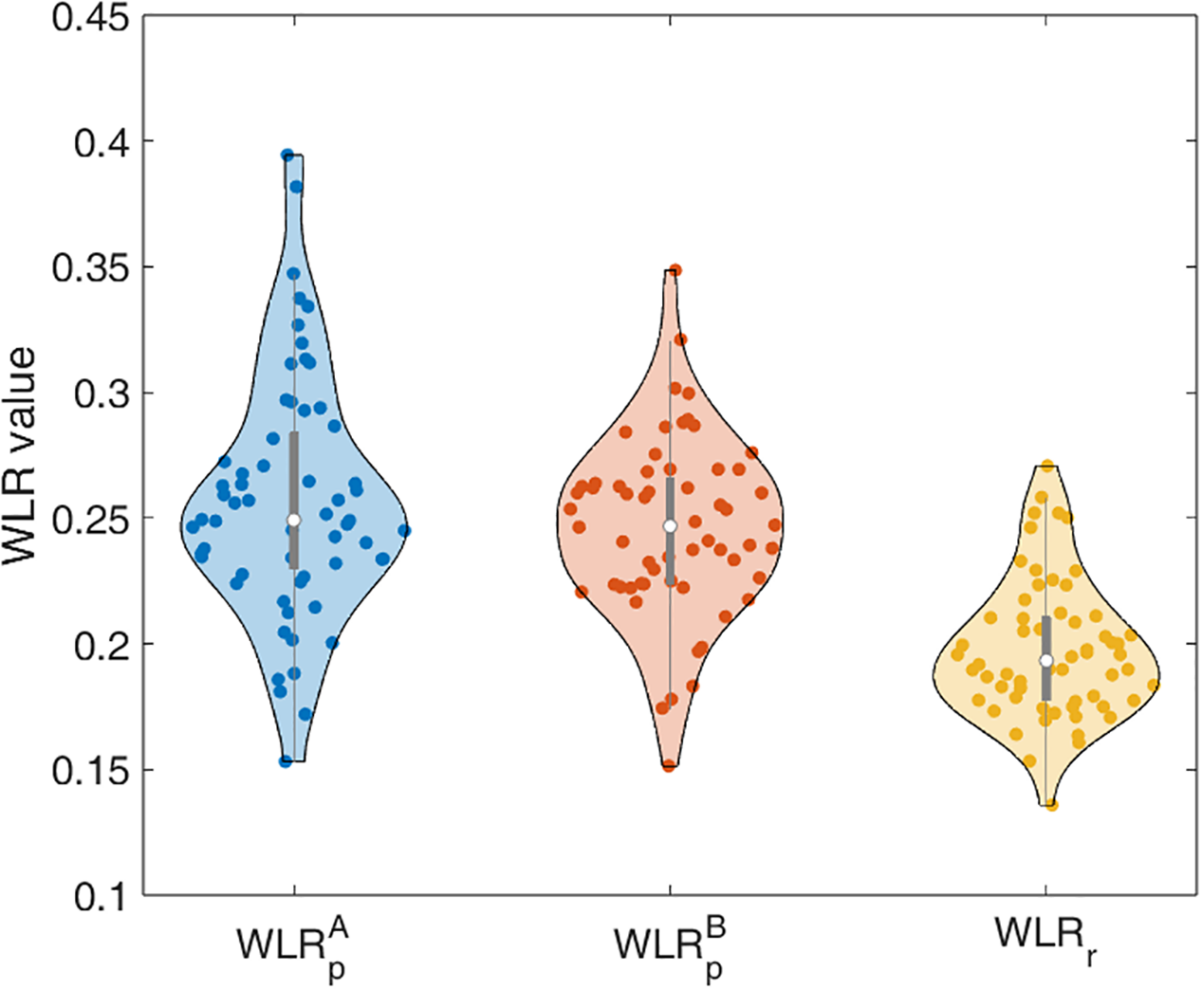
Violin plots showing the WLR distribution for the point-based approach for grader A, grader B, and for the region-based approach. As indicated, the point-based data for either grader presented with a WLR of higher magnitude compared to the region-based approach.

**Table 2.** Main Characteristics of WLR Values for Point-Based and Region-Based Evaluation

	Point-Based			Region-Based	
	Grader A	Grader B	Region-Based, Both Graders	Grader A, 30 images	Grader B, 30 images
Average	0.2570	0.2458	0.1971	0.2007	0.1936
Median	0.2492	0.2468	0.1933	0.1934	0.1901
Standard deviation	0.0483	0.0357	0.0257	0.0312	0.0246
Minimum value	0.1532	0.1514	0.1359	0.1359	0.1538
Maximum value	0.3944	0.3486	0.2747	0.2747	0.2588
Interquartile range	0.0543	0.0424	0.0332	0.0431	0.0293
Number of double-sided measurements	45	20	48	25	23

The characteristics for the region-based approach are described both for the entire dataset and separately for graders A and B.

The main statistical comparison of different approaches used is shown in Table 3 (comparison of two graders for the point-based approach) and in Table 4 (comparison of the point-based and region-based approaches).

**Table 3.** Comparison of Point-Based WLR Values for Grader A and B

Comparison	Value
Absolute/relative mean difference	0.0112/4.5%
Standard deviation of absolute differences	0.0207
Wilcoxon test	2.458 (*P* = 0.0108)
Intraclass Correlation Coefficient	0.721
Pearson Correlation Coefficient	0.778
Spearman Correlation Coefficient	0.780

**Table 4.** Comparison of Point-Based WLR Values With Region-Based WLR Values

Comparison	Grader A	Grader B
Absolute/relative mean difference	0.0608/26.1%	0.0495/19.9%
Standard deviation of absolute differences	0.0316	0.0210
Wilcoxon test	6.655 (*P* < 0.01)	6.706 (*P* < 0.01)
Intraclass Correlation Coefficient	0.301	0.358
Pearson Correlation Coefficient	0.750	0.840
Spearman Correlation Coefficient	0.752	0.793

#### Bland–Altman Analysis

WLR values were analyzed using Bland–Altman plots to visualize the agreement between the approaches or graders. We compared both individual graders within a point-based approach (Fig. 5, left panel) and we also compared point-based (see Fig. 5 middle graph: grader A; Fig. 5 right graph: grader B) and region-based approaches. Furthermore, we also show the relationship between the point-based and the region-based estimation for the 30 images from each grader, see Figure 6.

**Figure 5.**
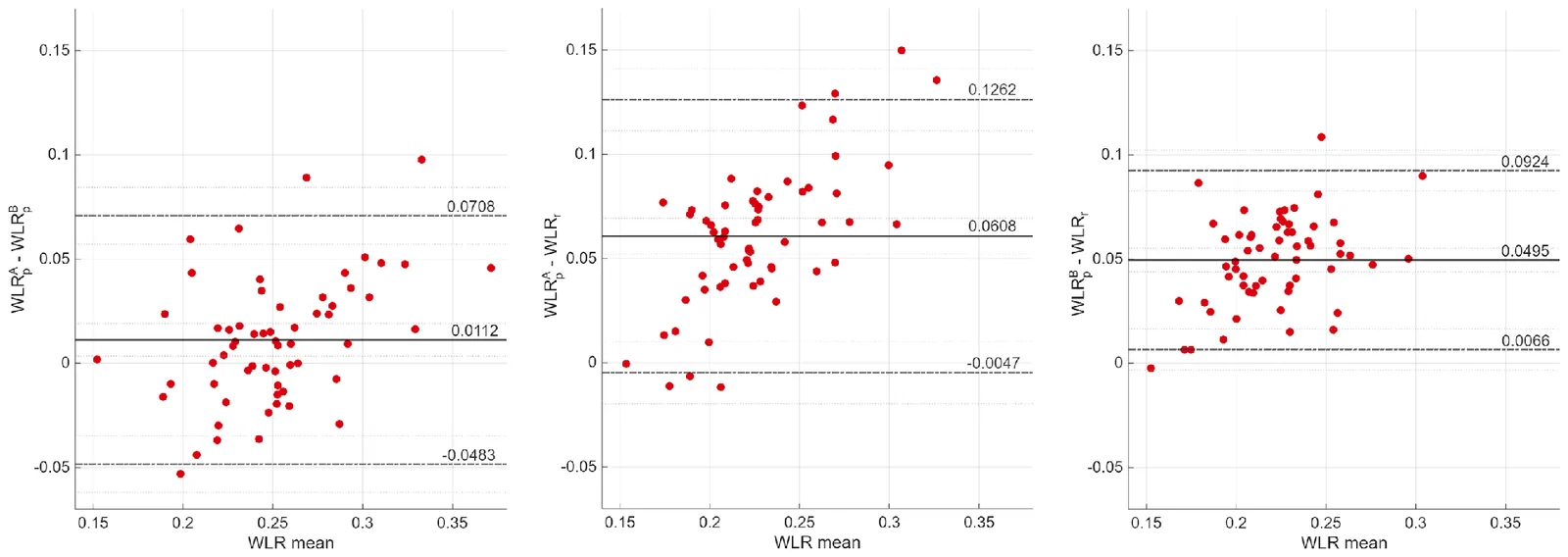
Bland–Altman plots. The differences between both graders for the point-based approach (*left* graph) and the difference between the point-based approach (*n* = 60, *middle* graph, grader A and *right* graph, grader B) and all gradings from the region-based approach (*n* = 60, i.e. *n* = 30 from grader A and *n* = 30 from grader). The analysis for the point-based approach ( WLR pA minus  WLR pB) demonstrates generally close agreement between graders. Regression of the difference on the pairwise mean yielded a slope of 0.3369 (*P* = 0.0004; intercept −0.0735), indicating statistically significant proportional bias. The Bland–Altman plots for the method comparison show wide range of agreement. The point-based approach provides systematically higher WLR values compared to the region-based approach (for both graders A and B, respectively), as indicated by the positive bias (WLR_p_ minus WLR_r_). The magnitude of this difference when compared to all region-based data tended to increase slightly with higher mean WLR values, especially for grader A (*middle* graph: grader A: slope = 0.6295, *P* < 0.001, intercept = −0.0819; and *right* graph: grader B: slope = 0.2964, *P* = 0.0014, intercept = −0.0160).

**Figure 6.**
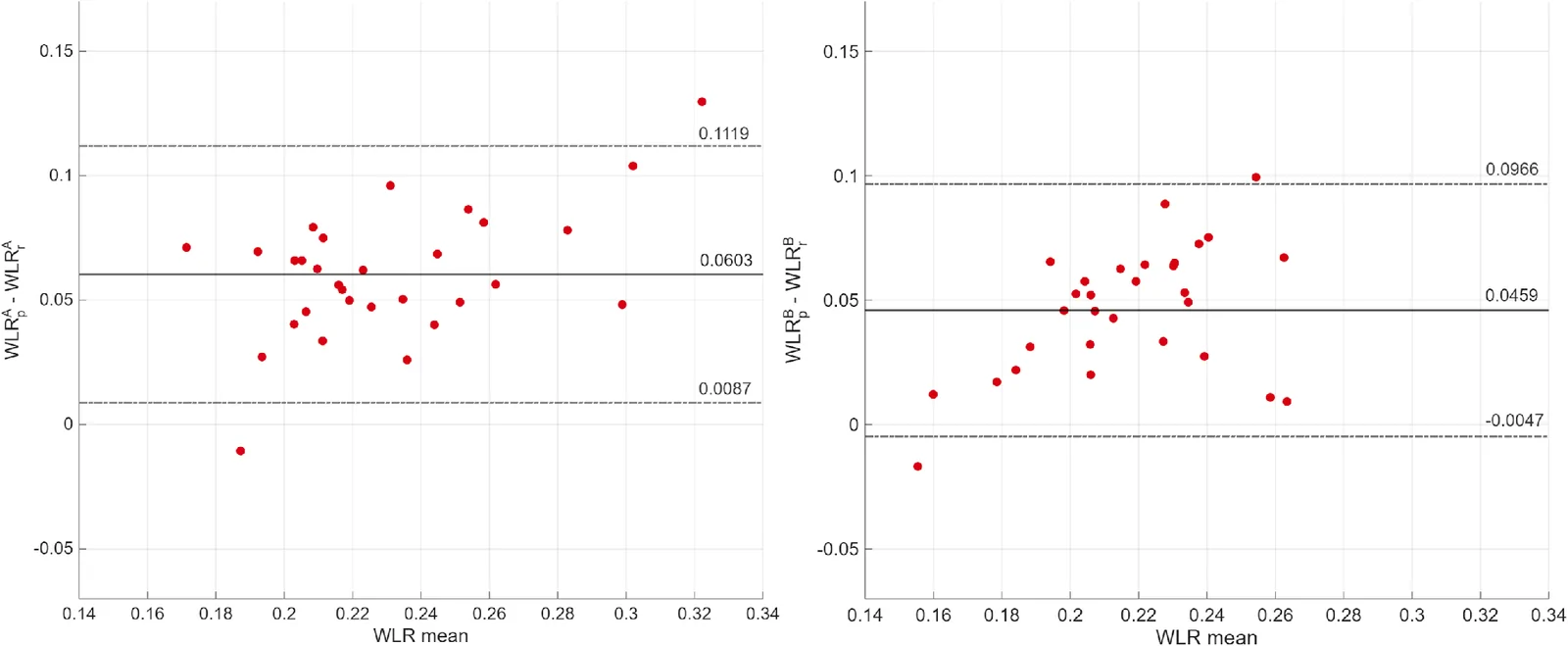
Bland–Altman plots showing the difference between the point-based approach versus the region-based approach for each grader, grader A (*left* graph: WLR^A^_p_ minus WLR^A^_r_)) and grader B (*right* graph: WLR^B^_p_ minus WLR^B^_r_), respectively. Here, half of the dataset was used for each plot, as the region-based approach was performed on 30 different images by each grader, respectively. The Bland–Altman plots for the method comparison show wide range of agreement. The point-based approach provides systematically higher WLR values compared to the region-based approach (for both graders A and B, respectively), as indicated by the positive bias (WLR_p_ minus WLR_r_). The magnitude of this difference, when compared to region-based data per grader, tended to increase slightly with higher mean WLR values (*left* graph: grader A: slope = 0.3774, *P* = 0.0037, intercept = −0.0268; *right* graph: grader B: slope = 0.4271, *P* = 0.0111, intercept = −0.0466).

Agreement between grader A and grader B for the point-based assessment of WLR (see Fig. 5, left panel) was evaluated using Bland–Altman analysis in 60 paired observations. The difference between the two graders was calculated for each observation, and the mean difference (WLR^A^_p_ minus WLR^B^_p_) was used to assess systematic bias. The mean difference was 0.0112 (95% CI = 0.0034–0.0191), indicating a small but statistically significant systematic difference between the graders (paired *t*-test: *t* = 2.866, *P* = 0.0058). The corresponding SD of the differences was 0.0304. The 95% limits of agreement (LoA) were −0.0483 to 0.0708, with 95% CIs of −0.0619 to −0.0347 for the lower limit and 0.0572 to 0.0844 for the upper limit, respectively. Thus, for approximately 95% of paired measurements, the difference between the 2 graders is expected to lie between −0.048 and 0.071 WLR units.

When comparing the point-based approach by grader A (*n* = 60) and the region-based approach for all (*n* = 60) assessments of WLR (see Fig. 5, middle panel), the difference (WLR^A^_p_ minus WLR_r_) between the two approaches was calculated for each observation, to assess systematic bias. The mean difference was 0.0608 (corresponding SD = 0.0334, 95% CI = 0.0521–0.0694), indicating a statistically significant systematic difference between the two grading approaches for grader A (paired *t*-test: *t* = 14.09, *P* < 0.001). As depicted in Figure 5, middle panel, the 95% LoA were −0.0047 to 0.1262, with 95% CIs of −0.0196 to 0.0103 for the lower limit and 0.1113 to 0.1412 for the upper limit, respectively. Thus, for approximately 95% of paired measurements, the difference between the point-based and region-based approaches is expected to lie between approximately −0.005 and 0.126 WLR units for grader A.

When comparing the point-based approach by grader B (*n* = 60) and the region-based approach for all (*n* = 60) assessments of WLR (see Fig. 5, right panel), the mean difference (WLR^B^_p_ minus WLR_r_) was 0.0495 (corresponding SD = 0.0219, 95% CI = 0.0439–0.0552), indicating a statistically significant systematic difference between the two grading approaches (paired *t*-test: *t* = 17.52, *P* < 0.001). As depicted in Figure 5, right panel, the 95% LoA were 0.0066 to 0.0924, with 95% CIs of −0.0032 to 0.0164 for the lower limit and 0.0826 to 0.1022 for the upper limit, respectively. Thus, for approximately 95% of paired measurements, the difference between the point-based and region-based approaches is expected to lie between approximately 0.007 and 0.092 WLR units for grader B.

To investigate agreement between the point-based gradings and the region-based gradings by grader A alone, WLR (see Fig. 6, left panel) was evaluated using Bland–Altman analysis in 30 paired observations. The mean difference was 0.0603 (corresponding SD = 0.0263, 95% CI = 0.0505–0.0701), indicating a statistically significant systematic difference between the two approaches (paired *t*-test: *t* = −12.54, *P* < 0.001). The 95% LoA were 0.0087 to 0.1119, with 95% CIs of 0.0949 to 0.1289 for the lower limit and 0.0083 to 0.0257 for the upper limit, respectively. Thus, for approximately 95% of paired measurements, the difference between the two grading approaches is expected to lie between approximately 0.009 and 0.112 WLR units.

To investigate agreement between the point-based gradings and the region-based gradings by grader B alone, WLR (see Fig. 6, right panel) was evaluated using Bland–Altman analysis in 30 paired observations. The mean difference was 0.0459 (95% CI = 0.0363–0.0556), indicating a statistically significant systematic bias between the two approaches (paired *t*-test: *t* = 9.73, *P* < 0.001). The corresponding SD of the differences was 0.0259. The 95% LoA were −0.0047 to 0.0966, with 95% CIs of 0.0799 to 0.1134 for the lower limit and −0.0215 to 0.0120 for the upper limit, respectively. Thus, for approximately 95% of paired measurements, the difference between the two grading approaches is expected to lie between approximately −0.005 and 0.097 WLR units.

#### Scatter Plots

The scatter plots (Fig. 7) visually clearly show the overall trend and systemic differences between both approaches and graders. These plots also highlight the correlation quantified in Table 4.

**Figure 7.**
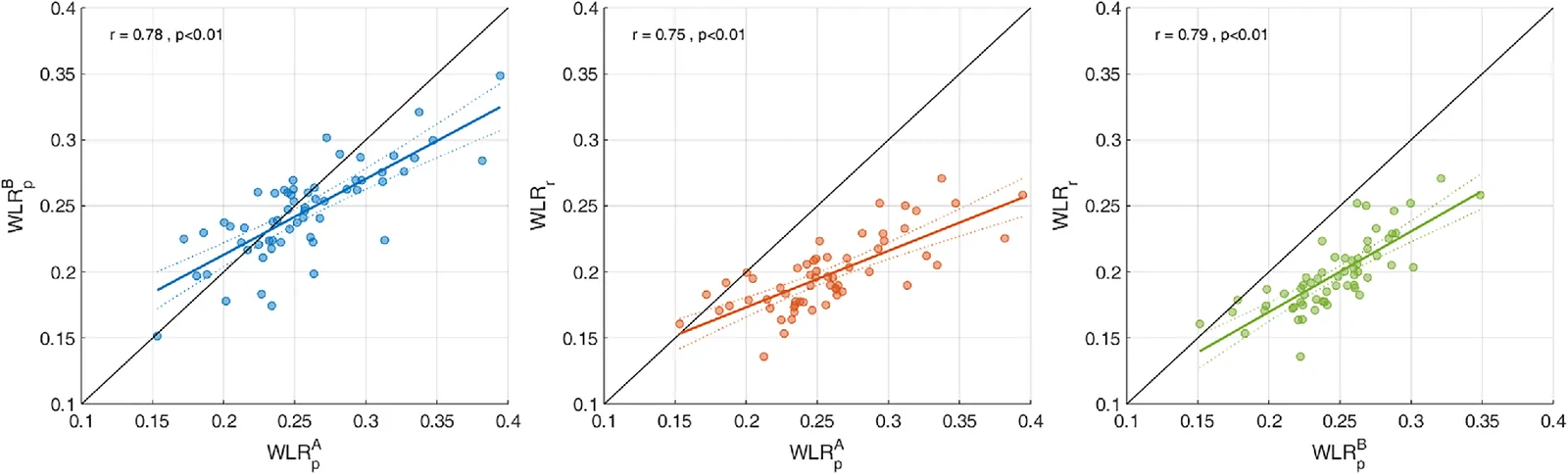
The correlation of both graders for the point-based approach is depicted in the *left* graph. When comparing grading by the point-based approach (grader A: *middle* graph and grader B: *right* graph) versus the region-based approach (*n* = 60) there is a tendency for lower WLR values for the region-based approach; the Spearman correlation is shown in the plots.

### Analysis of Point-Based Method

The 60 images of manual region-based segmentation were used to evaluate the dependence of the WLR differences on the number of points used to determine the WLR as described in the Methods section. This dependence is shown in Figure 8A for several specific images; and for the whole dataset in Figure 8B. In these graphs, each point represents one random sampling trial for a given number of sampling points (depicted on x axis). For each number of sampling points:

a) We examined 100 randomly generated positions, simulating various selection of sampling points.
b) For each of these hundreds of random positions, we computed relative error with respect to the WLR value estimated from all positions (named *WLR_global_*).
c) We plotted this relative error value into the graph as a single point.

**Figure 8.**
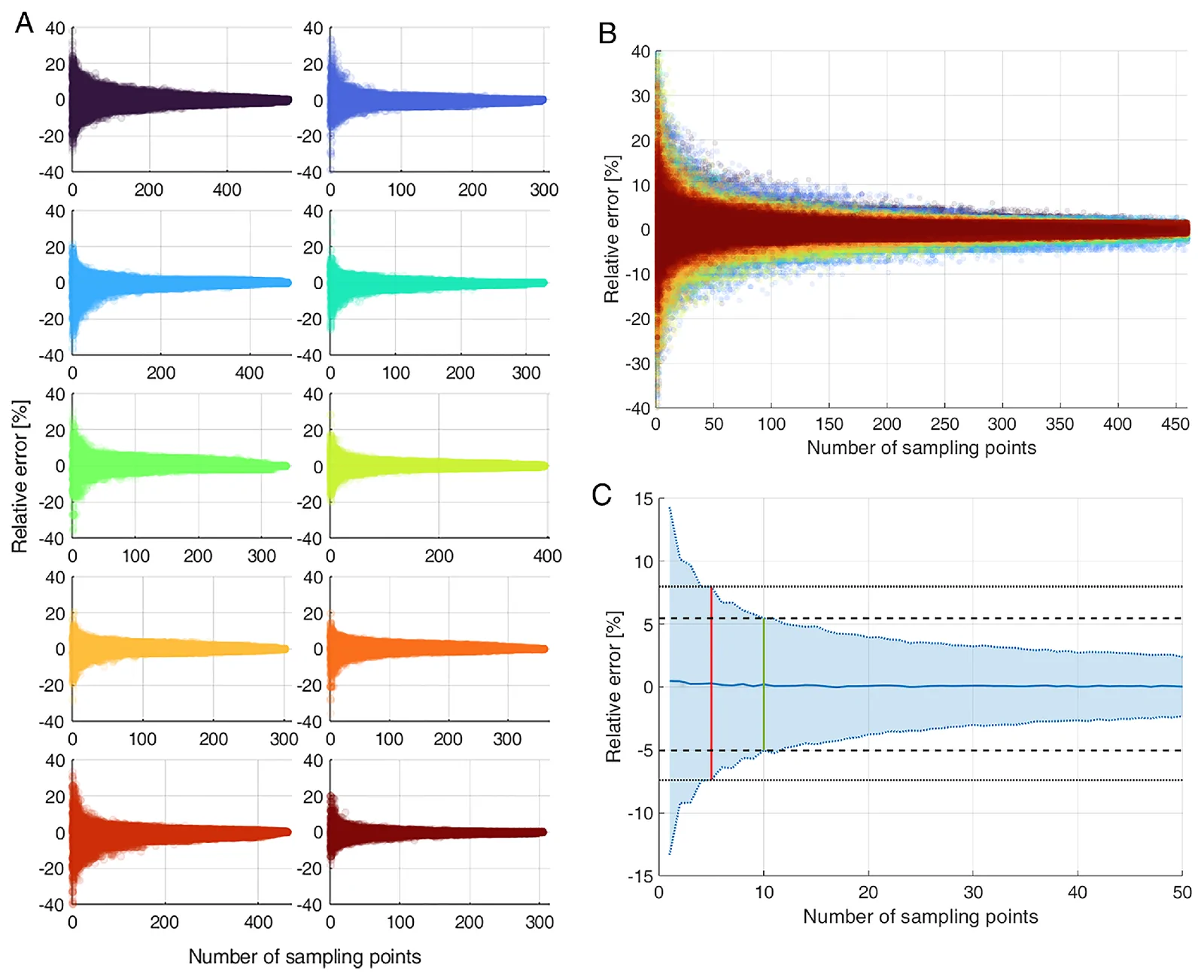
Plots showing the relative error obtained from random sampling. The graphs in (**A**) correspond to selected annotated images, whereas the graph (**B**) represents the results for the entire dataset, with each color indicating one image. The graph (**C**) shows the SD of the relative error across the whole dataset as a function of the number of sampling points. The two *vertical lines* indicate an example of 5 (*red* line) or 10 (*green* line) sampling points used for WLR computation, and the *dashed* and *dotted horizontal lines* denote the corresponding relative error levels. Therefore, 5 sampling points would lead to an error of approximately 8%, whereas 10 sampling points would lead to an error of 5%.

Thus, with an increasing number of sampling points, the relative error is decreasing, because we are approaching to *WLR_global_* value. Finally, the dependence of SD of this relative error on the number of sampling points *N* for the whole dataset is shown in Figure 8C.

## Discussion

There is growing interest in the in vivo assessment of retinal vessels, enabled by advances in AO-based imaging that provide rapid, high-resolution visualization of the retinal vasculature, including detailed views of the arterial wall and lumen important for detecting early vascular remodeling. Although WLR values can be influenced by various physiological and pathological factors (e.g. elevated blood pressure, smoking, obesity, and age), these effects were beyond the scope of this study and will be examined in future work. Our current analyses focused solely on the WLR measurement and how it differs depending on the estimation method. This focus highlights a broader challenge of ensuring that WLR extraction from retinal imaging data is performed in a repeatable and standardized manner. Current WLR studies predominantly rely on manual, point-based measurements, which are known to be strongly influenced by the grader's experience and by the number of sampling points used. The analysis presented in this work provides a comprehensive characterization of the WLR estimation error associated with these methodological factors.

As WLR is obtained from noninvasive imaging and assessment of a specific artery's lumen and wall therein, examination of WLR accuracy is difficult because the ground-truth WLR values of a specific eye are generally not known and cannot objectively be obtained. Manual WLR determination is the current clinical gold standard. As mentioned above, the experience of the grader, number of sampling points, as well as the image quality influences the measurement of WLR. For the point-based approach, our results present only a small difference between the graders, with a relative mean difference of only 4.5% for the whole dataset. These small inter-grader differences can be also observed from the Bland–Altman plot (see Fig. 5). Despite the large overlap in WLR values, a Wilcoxon test showed that there is a statistically significant difference between our two expert graders (*P* < 0.05). The scatter plot shows large correspondence pointing to high agreement between the graders (Pearson *r* = 0.778; Spearman *r* = 0.780; intraclass correlation coefficient = 0.721; see Table 3). For the region-based approach, inter-grader differences could not be investigated. However, the relative mean difference between the graders is 6.2%, which is comparable to the point-based method.

When investigating the difference between region-based and point-based WLR values, the relative mean difference is 26.1% and 19.9% for grader A or grader B, respectively. Supplementary Table S1 and Supplementary Table S2 investigate grader differences in evaluation of arterial lumen and wall.

Overall, the Bland–Altman analysis for the point-based approach demonstrates generally close agreement between graders (see Fig. 5, left panel). However, the magnitude of disagreement was not completely constant across the measurement range, indicating a proportional component to the inter-grader differences. Thus, although the absolute differences were small, the two graders cannot be considered fully interchangeable across the entire measurement range.

The Bland–Altman plots for the method comparison show the relatively wide range of agreement and the positive bias indicating that the point-based approach provides systematically higher WLR values compared to the region-based approach (for both grader A and B, respectively). The magnitude of this difference increased with the measurement level (see Fig. 6). Nevertheless, the observed differences generally fall within the 95% LoA. Thus, whereas the method comparison show systematic and proportional differences, the clinical relevance of this bias should be interpreted in relation to the expected magnitude and clinically acceptable range of WLR measurements.

The region-based approach allows evaluation across multiple positions to obtain a more complex picture about the influence of sampling points, as illustrated in Figure 8. The random sampling analysis revealed a strong dependence of WLR estimation error on the number of sampled positions. Ideally, to achieve a relative error of approximately 5% or less, approximately 10 sampling locations should be used to estimate WLR. Accepting a higher error tolerance reduces this requirement—for example, using 5 locations results in an error of approximately 8%. The relationship between relative error and the number of sampling points, shown in Figure 8C, provides a fundamental and practical insight into the estimation error that future studies should consider. It should be noted that the relative error in this figure represents the SD, and the actual error for a specific case may differ. This variability is also evident in Figure 8A, where error trends for individual images are presented. Based on our observations, these individual differences are primarily attributable to image quality, emphasizing that manual WLR assessment is inherently subjective and highly dependent on obtaining a sharp, well-defined vessel wall.

Furthermore, our observations indicate that the primary source of variability in WLR estimation arises from the subjective identification of vessel boundaries. In most cases, the uncertainty is greater when delineating the outer vessel wall, whereas, in some instances, difficulties also occur in defining the inner boundary (lumen), particularly when only one side of the vessel is visible. This effect is illustrated in Figure 9: the left image shows an example where both boundaries are well defined, whereas the middle and right images demonstrate cases where the inner border or central region of the artery is difficult to identify precisely, and where the sharpness of the wall–lumen structure varies spatially. Such inconsistencies primarily affect point-based approaches, in which individual boundary selections strongly influence the outcome, whereas the region-based method mitigates this effect by using the median value across multiple locations.

**Figure 9.**
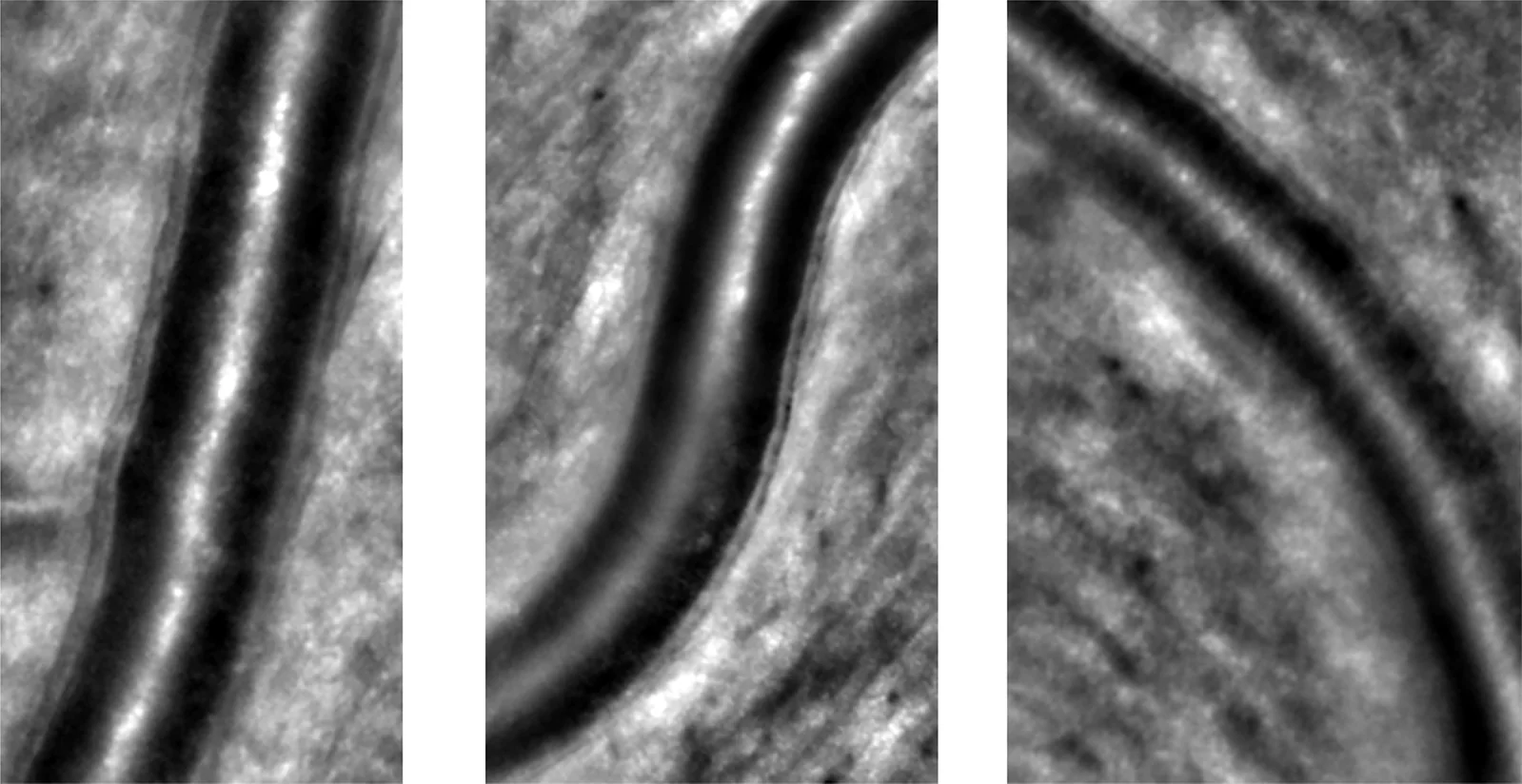
Examples of three magnified parts of the arteries. The *left* image represents a situation with clearly visible lumen and wall structure and their borders. The *middle* image represents situations with a sharp wall on one side only. The *right* image shows a situation where the inner border (lumen) is not clearly visible.

Possible variability in WLR estimates may arise from local and temporal caliber fluctuations of retinal arterioles, including vasomotion and other pulsatile changes, which can affect lumen diameter along the analyzed vessel segment in time-resolved image sequences. In our study, however, WLR was derived from static averaged images acquired with the rtx1 flood-illumination AO camera, so the impact of such transient fluctuations on the final analyzed image is expected to be negligible. Such potential influence was further reduced by excluding bifurcations and blurred regions and by aggregating measurements across multiple locations, which is consistent with our finding showing that sampling multiple points along the vessel improves the robustness of WLR assessment. Recent work by Senée et al.^21^ has shown that under nonstimulated baseline conditions, retinal arterial diameter exhibits only small, slow fluctuations, supporting the assumption that such effects are negligible within the short acquisition time and frame averaging used in our rtx1 protocol.

Although AO-based retinal imaging allows high-resolution visualization of small retinal vessels, its use for WLR assessment is subject to several limitations. These measurements are highly subjective and strongly influenced by the method used for WLR estimation. Consequently, accurate results depend heavily on the selection of arteriole regions and the overall quality of the AO images. Moreover, AO imaging is generally feasible only in individuals capable of maintaining steady fixation for extended periods, limiting its applicability in patients with certain ocular conditions. The technique is also susceptible to motion artifacts and is challenged by the low contrast between vessel walls and lumens.

## Conclusions

This study provides the first comprehensive comparison of two manual WLR estimation approaches in AO retinal images – point-based and region-based. This work highlights how sampling strategy drives estimation error. We show that the widely used point-based method is highly sensitive to measurement placement and obtaining ≥12 measurements locations per artery are needed to keep the relative error below 5%, offering a practical threshold for routine use. We also observed substantial discrepancies between the point-based and region-based approaches, likely arising from differences in how annotators segment data, how familiar they are with segmentation software and their experience level.

Based on our findings, we propose the following practical guidelines for manual WLR measurement in AO retinal images using the point-based method (typical in clinical practice):

1. Select only regions where the arteriolar wall and lumen boundaries are sharply visible and clearly distinguishable; avoid any locations where the vessel structure appears blurred or out of focus.
2. Perform double-sided measurements whenever both the inner and outer wall boundaries are recognizable on both sides of the vessel; use single-sided measurements only when one wall side is not sufficiently visible.
3. Distribute measurement locations along the entire usable length of the arteriolar segment to maximize spatial coverage and reduce the influence of local variability.
4. Avoid regions near vessel bifurcations or crossings, where local hemodynamics and anatomic complexity may bias wall and lumen thickness values.
5. Avoid locations where a neighboring vessel (artery or vein) runs in close proximity to the measured arteriole, as this may complicate boundary delineation and introduce measurement error.
6. Use at least 12 locations along the artery to measure WLR.
7. Remove the upper and lower decile of measured WLR values to remove potential outliers, that is, at least 10 individual WLR values should remain to keep the relative estimation error at approximately 5%.
8. Compute the median to obtain the final WLR value.

In summary, the variability and workload inherent to manual WLR estimation underscore the need for robust automated analysis methods. Such tools are essential for ensuring accurate, reproducible, and clinically applicable retinal microvascular assessments. However, these tools are not sufficiently developed or standardized, and there is a continuous need for research in this area with real clinical applicability, which motivates our future research.

## Supplementary Material

Supplement 1
