# Supplementary material for: Wall-to-Lumen Ratio Estimation in Adaptive Optics Retinal Imaging: Evaluation and Comparison
Source: Transl Vis Sci Technol. 2026 Oct 16;15(10):26. doi: 10.1167/tvst.15.10.26 (PMC13679783; doi:10.1167/tvst.15.10.26)
Supplement: Supplementary file 1 [file tvst-15-10-26_s001.pdf]

Supplement to

Wall-to-Lumen Ratio Estimation in Adaptive Optics Retinal Imaging: evaluation and comparison

Radim Kolar[1], Roman Jakubicek[1], Eva Orsagova[1], Jan Odstrcilik[1], Franziska G. Rauscher[2,3]

[1]*Department of Biomedical Engineering, Faculty of Electrical Engineering and Communication, Brno University of Technology, Brno, Czech Republic*

[2]*Institute for Medical Informatics, Statistics, and Epidemiology, Leipzig University, Leipzig, Germany*

[3]*Germany* Medical Informatics Center - Department of Medical Data Science, Leipzig University Medical Center, Leipzig, Germany

In this supplement, we provide example images with manual *point-based* annotation for two expert graders, to illustrate high (Table S1) and low (Table S2) inter-grader agreement. Graders were instructed to follow our standardized measurement protocol for manual point-based WLR assessment in AO retinal images. Graders were only allowed to select measurement locations where the arteriolar wall and lumen boundaries were sharply visible and clearly distinguishable, excluding blurred or out-of-focus regions. Then they were instructed to perform double-sided measurements whenever both the inner and outer wall boundaries were identifiable on both sides of the vessel and were allowed to use single-sided measurements only when one vessel wall was insufficiently visible. Graders needed to distribute the measurement locations along the entire usable length of each arteriolar segment to maximize spatial coverage while minimizing the influence of local variability. They were not allowed to make measurements near vessel bifurcations, crossings, and regions where adjacent arteries or veins were in close proximity to the target arteriole, to reduce potential bias in boundary delineation and wall-to-lumen estimation. We further stress that graders should obtain measurements at a minimum of 12 locations per vessel segment to maintain the relative estimation

error at approximately 5% during the analysis, where the upper and lower deciles of the measured WLR values were excluded and the median of the remaining measurements was calculated as the representative WLR for each vessel.

Table S1 and Table S2 present four example gradings by grader A and grader B, respectively.

Table S1. Examples of high inter-grader agreement. Each row contains one image evaluated by two graders, for grader A (first column), grader B (second column), with corresponding WLR values and relative difference stated (third column). In both cases, graders have independently evaluated almost the same positions. There is also high agreement regarding single-sided grading (orange dots) and double-sided grading (orange dots merged with blue dots). See lower row of examples, where grader A marked four single-sided positions in the same area as grader B, who marked three single-sided gradings. Likewise, a second area of the same artery, where grader A marked one double-sided position and grader B chose two double-sided gradings, however, since the positions are next to each other they produce high inter-grader agreement.

Grader A	Grader B	WLR values
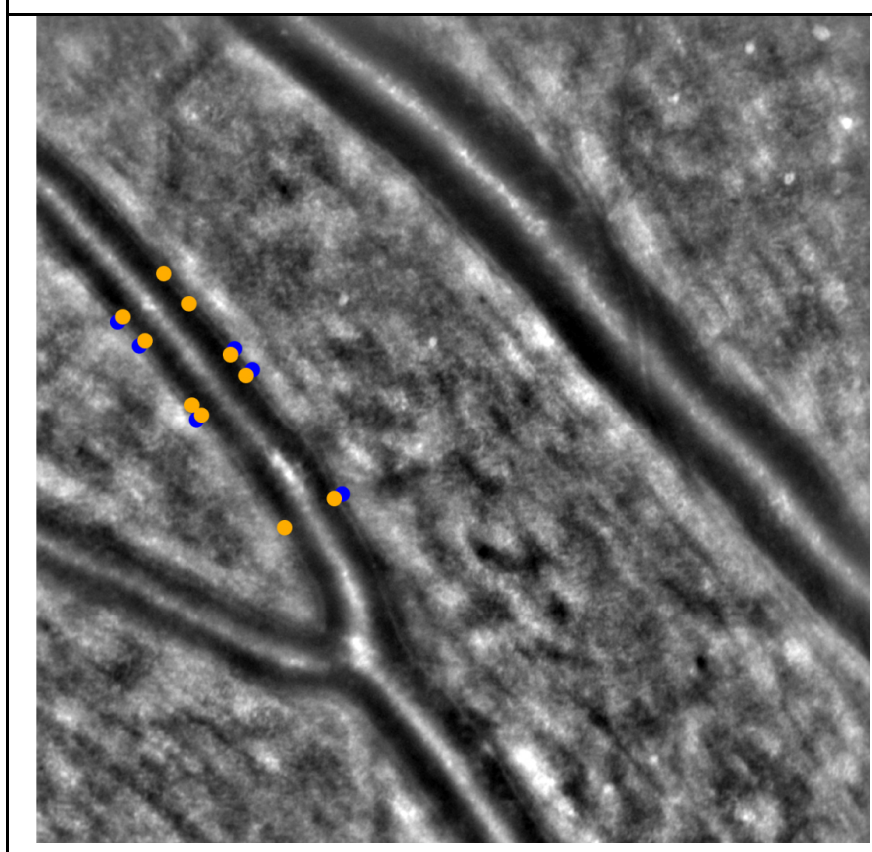	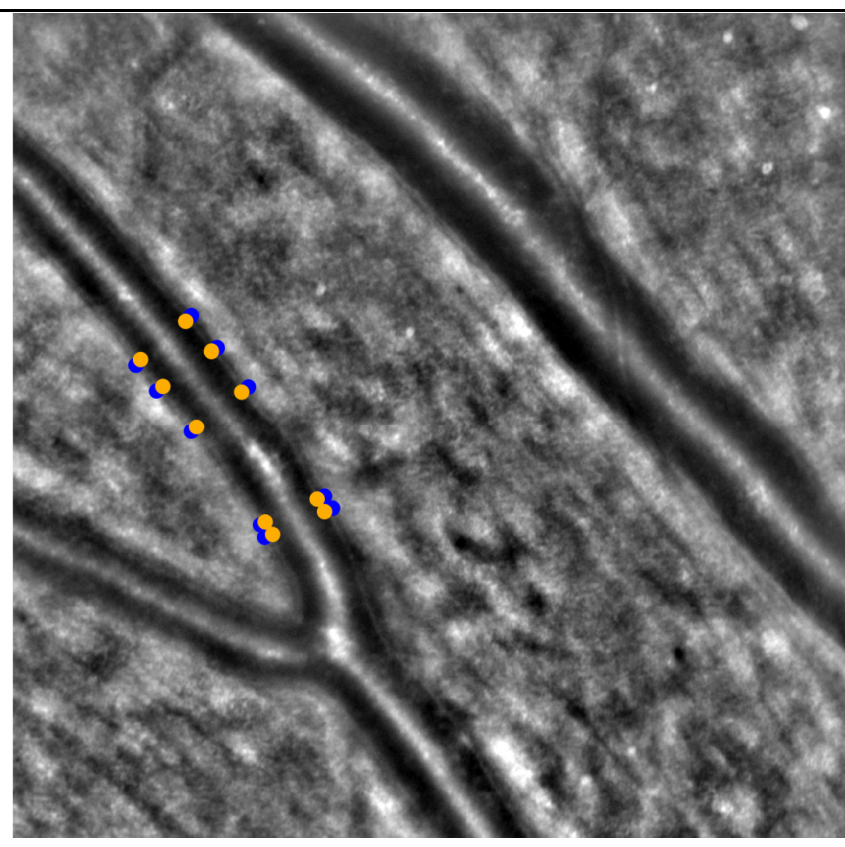	Grader A: 0.247<br><br>Grader B: 0.254<br><br>Relative difference: 2.8%
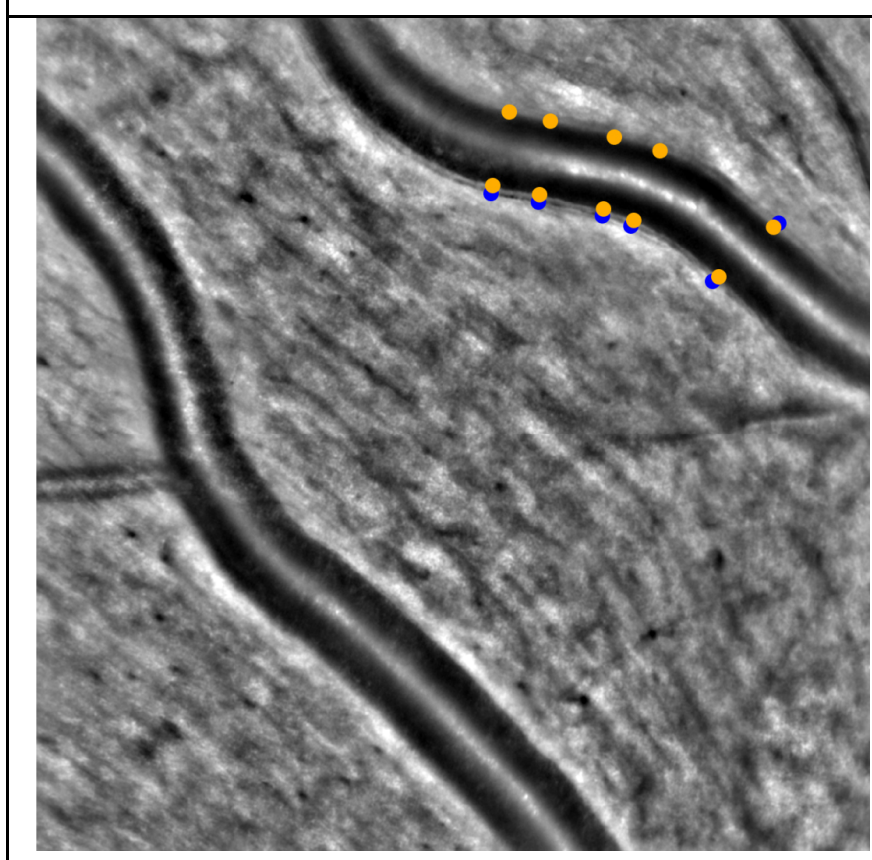	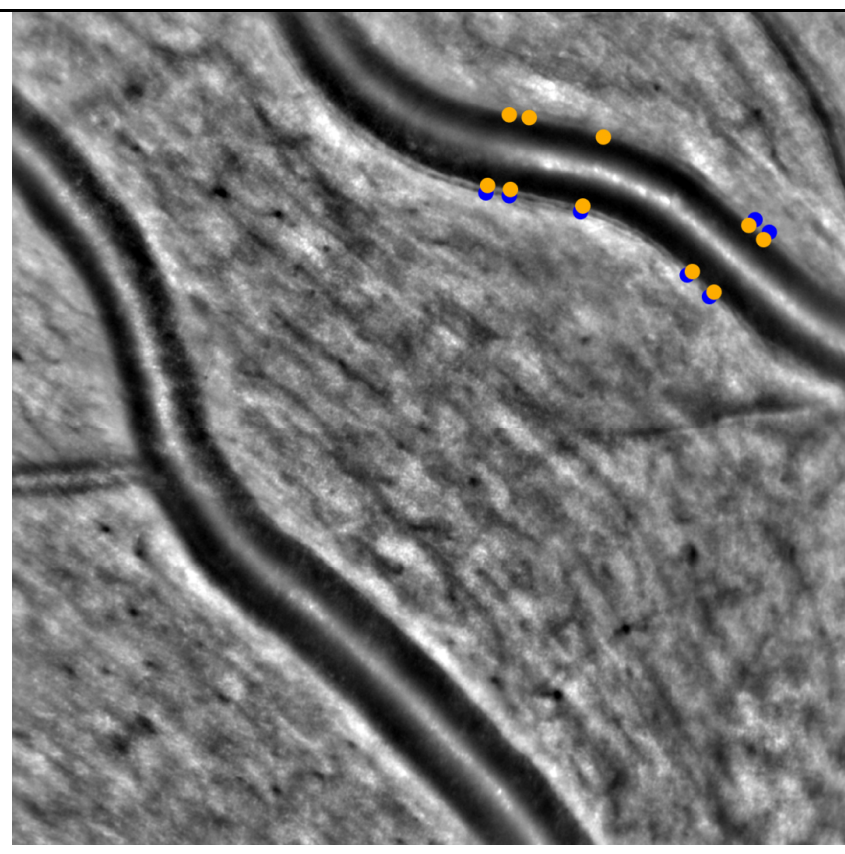	Grader A: 0.195<br><br>Grader B: 0.196<br><br>Relative difference: 0.5%

Table S2. Examples of low inter-grader agreement. Each row contains one image evaluated by two graders, for grader A (first column), grader B (second column), with corresponding WLR values and relative difference stated (third column). The lower agreement is likely due to differences between single-sided grading (orange dots) and double-sided grading (orange dots merged with blue dots) of the walls. In both rows, Grader A used mainly single-sided grading while Grader B used double-sided grading for the same image. Furthermore, both graders have evaluated WLR at slightly different positions in comparison to examples of Table S1 with high inter-grader agreement.

Grader A	Grader B	WLR values
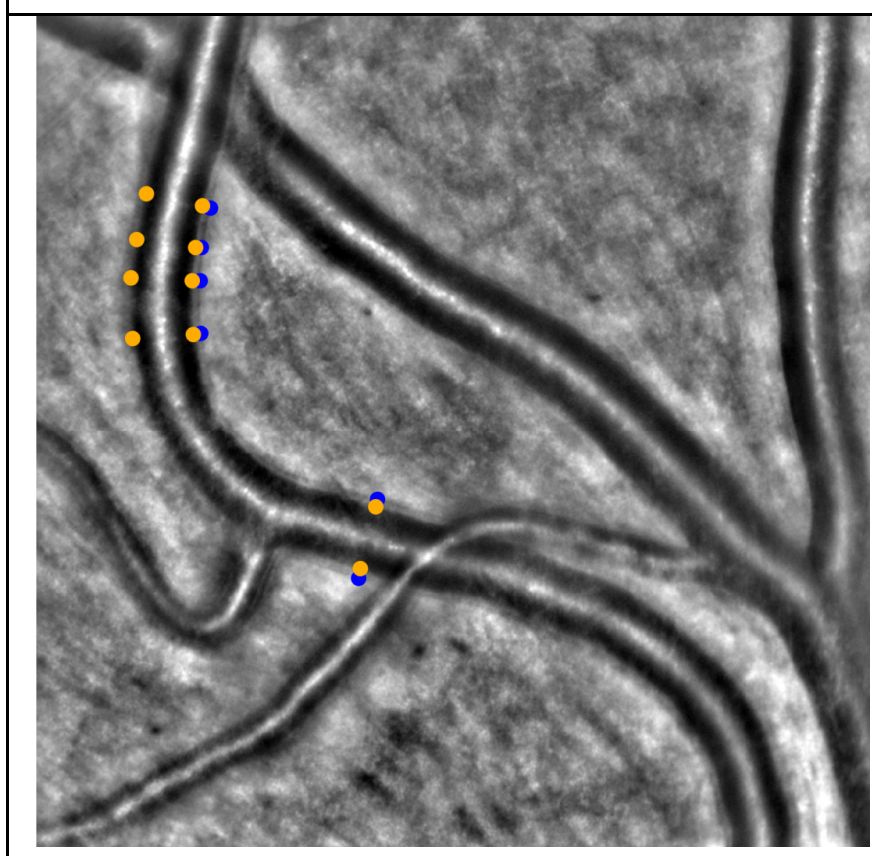	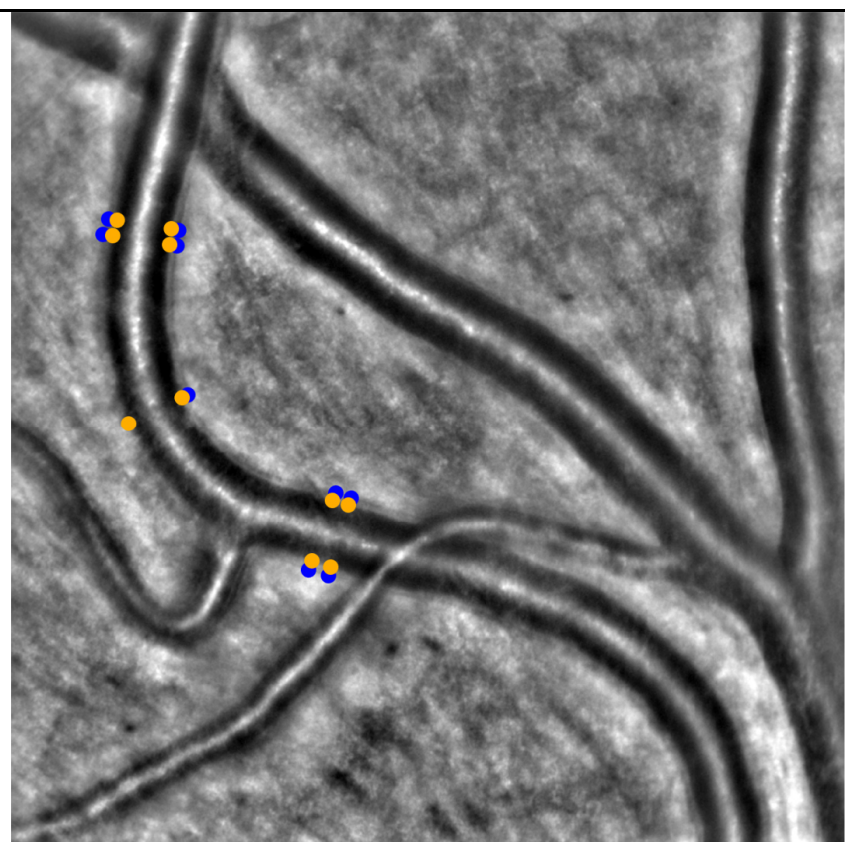	Grader A: 0.244<br>Grader B: 0.286<br>Relative difference: 15.9%
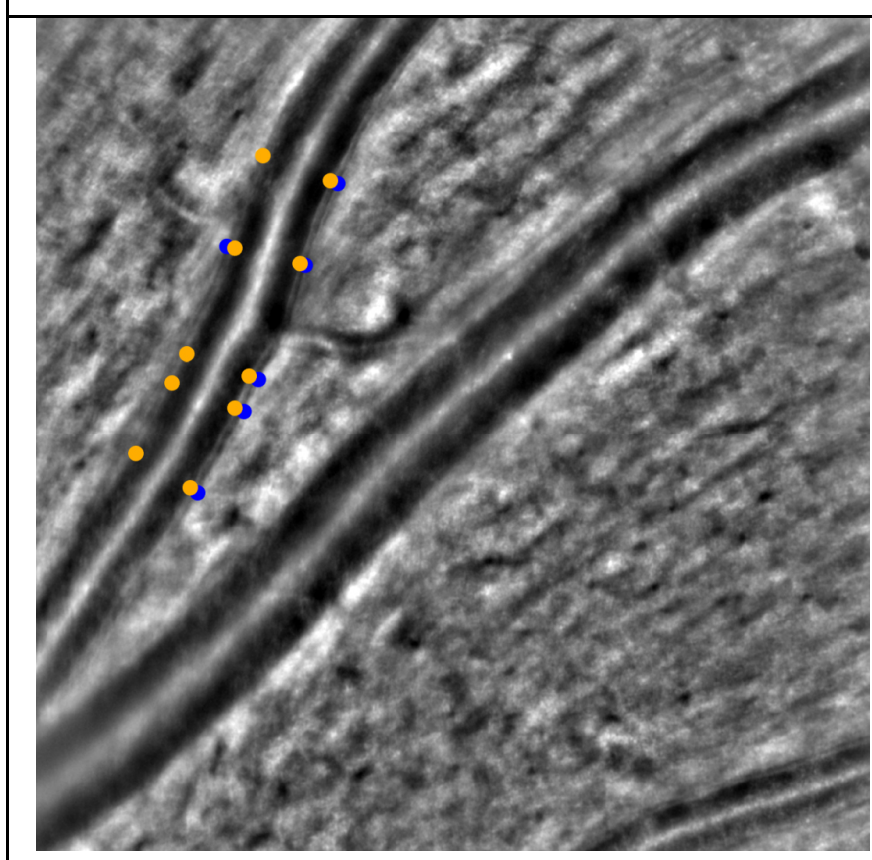	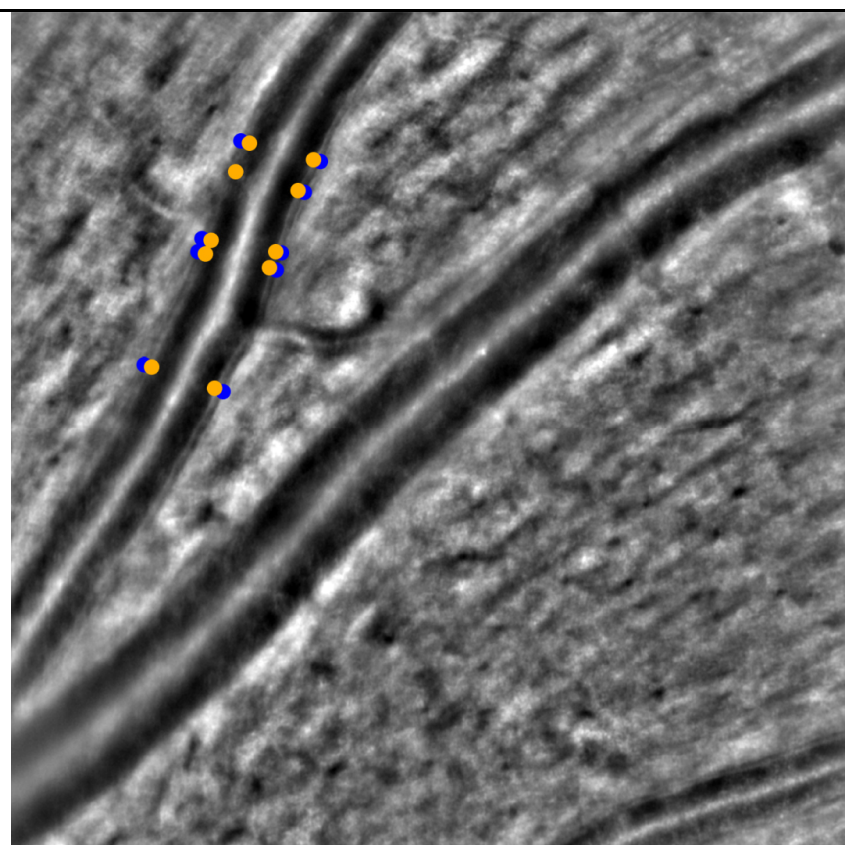	Grader A: 0.278<br>Grader B: 0.232<br>Relative difference: 18.0%
